# LncRNA X Inactive Specific Transcript Exerts a Protective Effect on High Glucose-Induced Podocytes by Promoting the Podocyte Autophagy via miR-30d-5p/BECN-1 Axis

**DOI:** 10.1155/2023/3187846

**Published:** 2023-03-03

**Authors:** Ying Cai, Sheng Chen, Xiaoli Jiang, Qiyuan Wu, Yong Xu, Fang Wang

**Affiliations:** ^1^Department of Nephrology, Ningbo Medical Center, Lihuili Hospital, Ningbo, China; ^2^Instrument R&D Center, Medical System Biotechnology Co., Ltd., Ningbo, China

## Abstract

Inhibiting podocyte autophagy promotes the development of diabetic nephropathy (DN). This study aims to explore the upstream regulatory mechanism of the autophagy-related gene BECN1 in high glucose (HG)-induced podocytes. C57BL/6 mice were treated with 50 mg/kg streptozotocin to construct a DN model. Biochemical indexes, pathological morphology of renal tissue, the morphology of renal podocytes, and the expressions of autophagy-related proteins in DN mice and normal mice were detected. The upstream miRNAs of BECN1 and the upstream long noncoding RNAs (lncRNAs) of miR-30d-5p were predicted by bioinformatics analysis and verified by dual-luciferase reporter assay. Mouse podocyte clone 5 (MPC5) cells were exposed to HG to construct a DN cell model. The levels of miR-30d-5p, X inactive specific transcript (XIST), and BECN1 in mouse kidney and MPC5 cells were detected by quantitative real-time polymerase chain reaction (qRT-PCR). The regulation of XIST/miR-30d-5p on the viability, apoptosis as well as proteins related to apoptosis, epithelial-mesenchymal transition (EMT), and autophagy in MPC5 cells were determined by rescue experiments. The levels of glucose, urinary protein, serum creatinine, and blood urea nitrogen were upregulated, but the kidney tissues and podocytes were damaged in DN mice. XIST targeted miR-30d-5p to promote viability while suppressing the apoptosis of HG-induced MPC5 cells. In kidney tissues or HG-induced MPC5 cells, the expressions of Beclin-1, light chain 3 (LC3) II/I, XIST, B-celllymphoma-2 (Bcl-2), and E-cadherin were downregulated, while the expressions of *P*62, miR-30d-5p, Bcl-2-associated X protein (Bax), cleaved-caspase-3, vimentin, and alpha-smooth muscle actin (*α*-SMA) were upregulated, which were reversed by XIST overexpression. The reversal effect of XIST overexpression was offset by miR-30d-5p mimic. Collectively, XIST promotes the autophagy of podocytes by regulating the miR-30d-5p/BECN1 axis to protect podocytes from HG-induced injury.

## 1. Introduction

Diabetic nephropathy (DN) is the main cause of end-stage renal disease (ESRD) worldwide, and hyperglycemia is the main factor driving DN into ESRD [1]. The typical symptom of DN is proteinuria, and podocyte injury is closely related to proteinuria [2]. Podocytes are highly differentiated terminal glomerular epithelial cells, which play an important role in regulating glomerular function. Podocyte injury is the basic feature of glomerular diseases, including podocyte fusion, podocyte hypertrophy, podocyte number reduction, podocyte apoptosis, podocyte epithelial-mesenchymal transition (EMT), and so on [3]. Therefore, protecting podocytes from high glucose (HG)-induced injury may be a new strategy to treat DN.

Increasing research has shown that the deficiency of autophagy is the key cause of podocyte injury [4, 5]. Autophagy is the process of transporting damaged organelles and aging proteins in cells to lysosomes for degradation [6]. Therefore, podocytes need to maintain intracellular homeostasis through autophagy, and the lack of autophagy will lead to podocyte injury, proteinuria, and glomerulosclerosis [5]. Beclin-1 encoded by BECN1 is an indispensable protein in autophagy and is the key factor of autophagy initiation. The activation of autophagy mediated by Beclin-1 is an important mechanism to reduce podocyte injury induced by HG [7]. Therefore, it is our focus to promote the autophagy of podocytes by activating the expression of Beclin-1 in podocytes.

Epigenetic mechanism exerts an important effect on the regulation of malignant tumors, immune diseases, and metabolic diseases [8]. Noncoding RNA, an epigenetic mechanism, including long noncoding RNA (lncRNA) and microRNA (miRNA), has been proven to be involved in the development of DN and has been suggested as a diagnostic marker or therapeutic target for DN [9, 10]. miRNA can inhibit the translation of mRNA or promote its degradation by binding to downstream mRNA [11]. Through an online database, in this research, the possible target miRNAs of the BECN1 gene were predicted, among which miR-30d-5p has been proven to suppress the autophagy of renal cell carcinoma cells [12]. Nevertheless, whether miR-30d-5p is also involved in the regulation of podocyte autophagy is yet to be elucidated, which is thus discussed in this research. Given that lncRNAs often participate in the disease progression by sponging miRNA to inhibit the function of miRNA, the upstream lncRNAs of miR-30d-5p were predicted in this research, of which lncRNA XIST was reported to participate in the protection of podocytes against HG-induced cell injury by regulating the miR-30/AVEN axis [13]. Therefore, we speculated that XIST may engage in the modulation of podocyte autophagy via the miR-30d-5p/BECN1 axis, thereby exerting a protective effect on HG-induced podocytes.

## 2. Materials and Methods

### 2.1. Animals and Drug Administration

C57BL/6 mice (*n* = 10, 18–22 g) were supplied by Shanghai Slake Animal Laboratory Co. Ltd. The room temperature was maintained at 22 ± 2°C and humidity was set at 50–60% with a normal period (12 hours (h) light/12 h dark). The research was approved by the Ethics Committee of Zhejiang Baiyue Biotech Co., Ltd. for Experimental Animals Welfare (approval number: ZJBYLA-IACUC-20210818).

The mice were randomly divided into two groups, namely, the DN group (*n* = 5) and the control group (*n* = 5). Subsequently, the DN model was constructed as previously described [14]. Briefly, mice in the DN group were treated with 50 mg/kg streptozotocin (STZ, S0130, Merck, Germany) in citrate buffer (C2488, Merck, Germany) for 5 consecutive days, and those merely treated with citrate buffer were applied as the control group.

### 2.2. Biochemical Parameters and 24 h Urine Protein Determination

Blood samples were collected from the tail of mice, and glucose (GLU) was tested every week by a blood glucose meter (Accu-Chek, Roche, Switzerland) to ensure the successful construction of the diabetes model. Four weeks after the injection of STZ, the 24-h urine of mice was collected, and the urine protein was determined by the urine protein test kit (C035-2-1, Nanjing Jiancheng, China). The establishment of the DN model was considered to be successful, as obvious albuminuria was detected in mice. Blood from the tail vein of mice was collected and centrifuged at 7000 r/min for 10 minutes at 4°C to obtain plasma samples. The levels of serum creatinine (Scr) and blood urea nitrogen (BUN) in plasma were detected by an automatic biochemical analyzer (AU680, Beckman, USA).

### 2.3. Histopathological Examination

The mice were sacrificed by cervical dislocation and their renal tissues were collected and fixed in 4% paraformaldehyde. The fixed kidney tissues were embedded in paraffin and made into 4 *μ*m sections. After being dewaxed with xylene and rehydrated with gradient ethanol, the sections were subjected to staining with the hematoxylin and eosin (H&E) kit (G1003, Servicebio, China) and Periodic Acid-Schiff (PAS) staining kit (G1008, Servicebio, China), respectively. Thereafter, the sections were dehydrated and transparentized. Finally, the sections were sealed with glycerol gelatin aqueous (S2150, Solarbio, China), and the results were observed under a microscope (AE2000, Motic, China).

### 2.4. Ultrastructural Changes under Transmission Electron Microscope

The renal tissues were washed in phosphate-buffered saline (PBS) and then fixed in a fixing solution (G1102, Servicebio, China). After dehydration by acetone, the tissues were embedded in resin and sectioned with an ultrathin microtome (Leica EM UC7, Germany). The sections were stained with uranyl acetate and lead citrate, and the morphological changes of renal podocytes were observed by the transmission electron microscope (HITACHI HT7700, Japan).

### 2.5. Immunohistochemistry

The sections were routinely dewaxed and hydrated and then were subjected to antigen repair using antigen repair solution (G1202, Servicebio, China). After being treated with 3% H_2_O_2_, the sections were blocked with bovine serum albumin (BSA; abs9157, Absin, China) and then probed with the primary antibody against light chain 3B (LC3B; ab63817, Abcam, USA) and horseradish peroxidase (HRP)-labeled secondary antibody goat anti-rabbit IgG (S0001, Affinity, USA). Thereafter, LC3 expression was visualized by 3,3′-diaminobenzidine (DAB) solution (G1212, Servicebio, China), and the nuclear staining was conducted by a hematoxylin staining solution. Finally, the results were observed under the microscope.

### 2.6. Western Blot

Kidney tissues or mouse podocyte clone 5 (MPC5) cells were subjected to the extraction of total proteins using the protein extraction kit (W034, Nanjing Jiancheng, China). Next, the protein concentration was quantified with the BCA protein assay kit (*P*1511, Applygen, China). Subsequently, the proteins were separated by SDS-PAGE gel (W003, Nanjing Jiancheng, China), blocked with BSA, and successively incubated using primary antibodies and secondary antibodies. The details of the antibodies are shown in Table 1. Glyceraldehyde-3-phosphate dehydrogenase (GAPDH) served as an internal reference. Eventually, the protein bands were visualized using an electrochemical luminescence reagent (W028, Nanjing Jiancheng, China) with a Tanon 5200 Imaging System (Shanghai, China).

### 2.7. Bioinformatics Assay

The miRDB (https://mirdb.org/index.html) and TargetScan (https://www.targetscan.org/vert_72/) were used to search the miRNAs targeting BECN1. The binding sites between miR-30d-5p and BECN1/XIST were predicted by TargetScan and Starbase (https://starbase.sysu.edu.cn/).

### 2.8. Cell Culture

Mouse podocytes MPC5 (BNCC342021, BeNa Culture Collection, China) were cultivated in DMEM-H complete medium (BNCC338068, BeNa Culture Collection, China) at 37°C with 5% CO_2_. For glucose exposure, MPC5 cells were treated with normal glucose (NG, 5.5 mmol/L) or HG (30 mmol/L) for 48 h as previously described [15].

### 2.9. Transfection

MiR-30d-5p mimic (M, miR10000515-1-5) and mimic control (MC, miR1N0000002-1-5) were obtained from Ribobio (China). The gene sequence of XIST was obtained from the NCBI database and then amplified by polymerase chain reaction (PCR). The amplified gene sequence was inserted into pCDNA3.1–3 × FLAG-N (ZL-3.1FLAGN, Ke Lei Biological Technology, China) to construct the XIST overexpression (OE) plasmid (hereafter called OE-XIST). MPC5 cells were inoculated into a six-well plate and cultured to be approximately 80% confluent prior to transfection. Thereafter, OE-XIST/empty vector or miR-30d-5p mimic/mimic control was transfected into cells by Lipofectamine 2000 Transfection Reagent (11668-019, Invitrogen, USA) that had been diluted in Opti-MEM™ medium (31985070, Thermo Fisher, USA). After being incubated for 24 h, the transfected cells were analyzed by quantitative real-time PCR (qRT-PCR).

### 2.10. Target Gene Verification

The target gene verification was conducted by dual-luciferase reporter assay. MiR-30d-5p mimic/mimic control and the pmirGLO vector (E1330, Promega, USA) containing the wild-type (WT) or mutant (MUT) 3′UTR region of BECN1 or XIST were cotransfected into 293T cells (BNCC353535, BeNa Culture Collection, China) using Lipofectamine 2000 Transfection Reagent. Post 48 h transfection, the activities of firefly luciferase and Renilla luciferase were measured using the Dual-Luciferase® Reporter Assay System (E1910, Promega, USA).

### 2.11. qRT-PCR

Total RNA was extracted from MPC5 cells using Trizol reagent (15596026, Invitrogen, USA) and miRNeasy mini kit (217004, Qiagen, German). The cDNA synthesis was performed using the first strand cDNA synthesis kit (K1612 or B532453, Sangon, China). The qPCR was conducted on a real-time PCR system (CFX Connect, Bio-rad, USA) using SYBR Green Abstract PCR Mix (B110031) and MicroRNAs qPCR Kit (B532461) obtained from Sangon (China). The relative values were calculated by the 2^−ΔΔCT^ method [16] and normalized to GAPDH or U6. The sequences of the primers are listed in Table 2.

### 2.12. Cell Counting Kit-8 (CCK-8) Assay

MPC5 cells in the logarithmic phase were harvested and prepared into 3 × 10^4^/mL cell suspension, followed by being added into a 96-well plate (2 × 10^3^ cells/well). After the cells were incubated with 10 *μ*L CCK-8 solution (C0005, TopScience, China) for 4 h, the absorbance at 450 nm was determined with a microplate reader (CMaxPlus, MD, China).

### 2.13. Cell Apoptosis Assay

Annexin V-FITC/PI Apoptosis Detection Kit (556547, BD, USA) was utilized to evaluate the cell apoptosis. MPC5 cells were digested and prepared into 1 × 10^6^/mL cell suspension in binding buffer, and the treated cell suspension was incubated with 5 *μ*L of Annexin V-FITC and 10 *μ*L of *PI* *for* 20 minutes in the dark. In the end, the cell apoptosis was detected by an Accuri C6 flow cytometer (BD Biosciences, USA).

### 2.14. Statistical Analysis

The measurement data were presented as mean ± standard deviation. The results between the two groups were analyzed by an independent sample *t* test. One-way analysis of variance (ANOVA) was adopted for the comparison among multiple groups. The experiments were repeated three times. All statistical analyses were implemented with GraphPad 8.0 software, and *P* value less than 0.05 was considered to be statistically significant.

## 3. Results

### 3.1. Autophagy Inhibition was Observed in the Kidney Tissue of DN Mice

**Figure 1:**
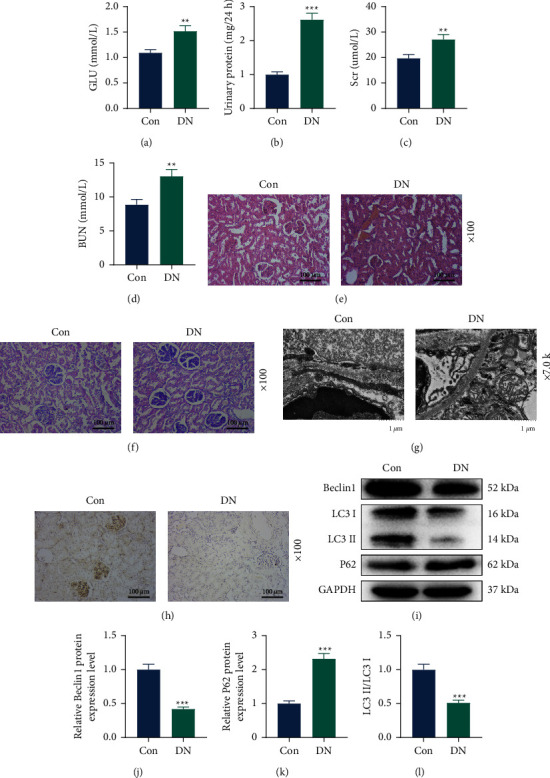
Detection of biochemical indexes, pathological changes, and autophagy-related proteins in DN mice and normal mice. C57BL/6 mice were treated with 50 mg/kg STZ in citrate buffer (DN group) for 5 consecutive days or treated with citrate buffer (control group). (a) The whole-blood GLU was measured using a blood GLU meter. (b) The urinary protein concentration was determined by the corresponding kit. (c) and (d) SCR and BUN levels were detected using an automatic biochemical analyzer. (e) Hematoxylin and eosin staining of kidney tissues (magnification: 100x times; scale bar = 100 *μ*m). (f) PAS staining of kidney tissues (magnification: 100x times; scale bar = 100 *μ*m). (g) Renal podocytes were observed by a transmission electron microscope (magnification: 7000x times; scale bar = 1 *μ*m). (h) Immunohistochemistry was used to detect the expression of LC3 in kidney tissues (magnification 100x times; scale bar = 100 *μ*m). (i)–(l) The protein levels of Beclin 1, LC3 I/II, and *P*62 in kidney tissues were detected by Western blot. GAPDH was used as the internal control. Quantified values were presented as mean ± standard deviation of at least three independent experiments. ^*∗*^*p* < 0.01, ^*∗∗*^*p* < 0.001 vs. control. STZ: streptozotocin. DN: diabetic nephropathy. GLU: glucose. SCR: serum creatinine. BUN: blood urea nitrogen. GAPDH: glyceraldehyde-3-phosphate dehydrogenase. PAS: periodic acid Schiff.

We detected the levels of GLU, urinary protein, Scr, and BUN in two groups of mice, and found that the levels of these indexes in DN mice were higher than those in control mice (Figures 1(a)–1(d), *P* < 0.01). Histological staining results showed that compared with those of the control mice, the glomerular structure of DN mice was disordered and the basement membrane was thickened (Figures 1(e) and 1(f)). Through transmission electron microscope, we observed the disorganized podoid processes, exfoliated podocytes, and thickened basement membrane in DN mice (Figure 1(g)). In addition, the expression of LC3 in the kidney tissue of DN mice was decreased (Figure 1(h)). Meanwhile, the results of Western blot showed that the levels of Beclin1 and LC3 II/LCE I were lower yet the *P*62 level was higher in the DN group than those in the control group (Figures 1(i)–1(l), *P* < 0.001).

### 3.2. MiR-30d-5p Targeted BECN1 and XIST

**Figure 2:**
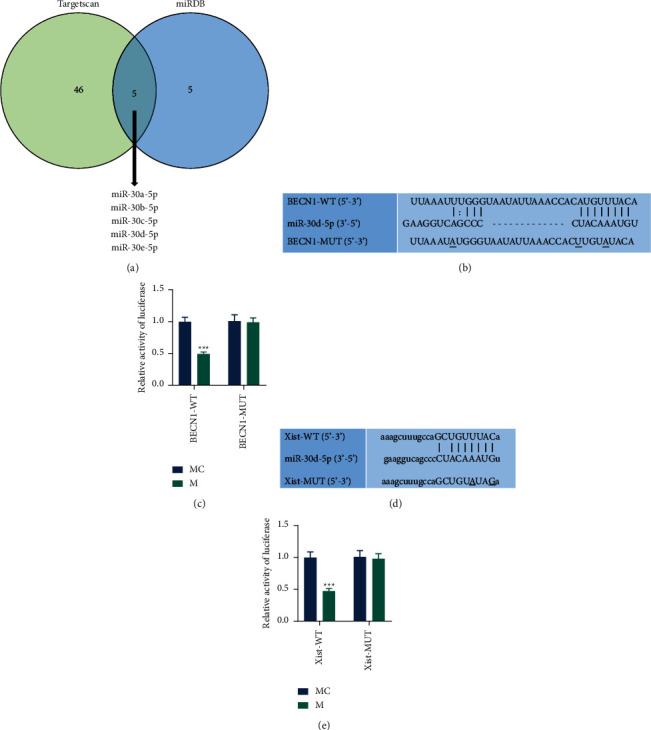
Validation of the targeting relationship of XIST/BECN1 and miR-30d-5p. (a) The miRDB (https://mirdb.org/index.html) and TargetScan (https://www.targetscan.org/vert_72/) were used to search the miRNAs targeting BECN1. The results were intersected by a Venn diagram. (b) The binding sites between miR-30d-5p and BECN1 were predicted by TargetScan. (c) The relationship between miR-30d-5p and BECN1 was verified by dual-luciferase reporter assay. (d) The binding sites between miR-30d-5p and XIST were predicted by Starbase (https://starbase.sysu.edu.cn/). (e) The relationship between miR-30d-5p and XIST was verified by dual-luciferase reporter assay. Quantified values were presented as mean ± standard deviation of at least three independent experiments. ^*∗∗*^*p* < 0.001 vs. MC. qRT-PCR: quantitative real-time polymerase chain reaction. GAPDH: glyceraldehyde-3-phosphate dehydrogenase. PAS: periodic acid Schiff reaction. DN: diabetic nephropathy. M: mimic. MC: mimic control; XIST: X inactive specific transcript.

We predicted the possible target miRNAs of BECN1 through the online website and then obtained five candidate miRNAs by the intersection of the results (Figure 2(a)). Based on the literature retrieval results, miR-30d-5p was finally selected as the target miRNA of BECN1 and XIST for subsequent studies. According to the predicted sequences in Figures 2(b) and 2(d), we construct the recombinant plasmid and transfect it with miR-30d-5p mimic into 293T cells. The results indicated that the luciferase activity of 293T cells transfected with miR-30d-5p mimic and BECN1-WT plasmid or XIST-WT was reduced (Figures 2(c) and 2(e), *P* < 0.001).

### 3.3. XIST Targeted miR-30d-5p to Regulate the Viability and Apoptosis of MPC5 Cells

**Figure 3:**
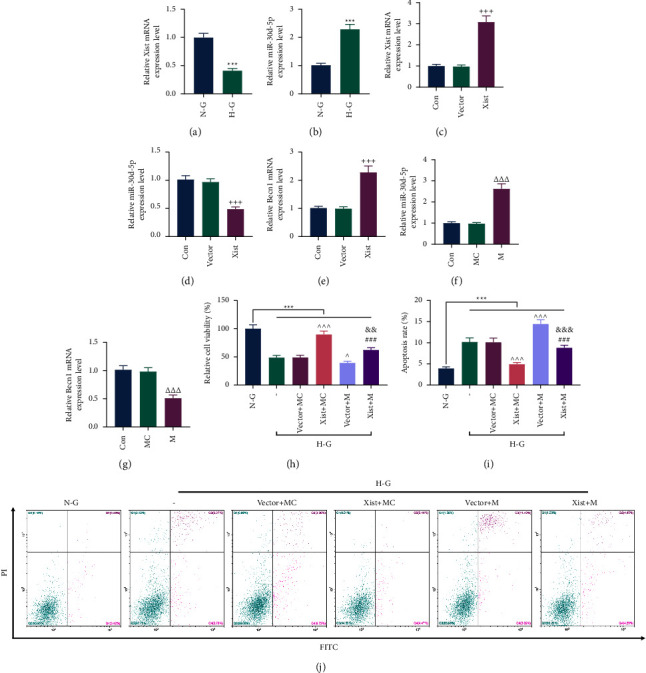
XIST targeted miR-30d-5p to regulate the viability and apoptosis of HG-induced podocytes. MPC5 cells were transfected with XIST overexpression plasmid/empty vector and or miR-30d-5p mimic/mimic control. (a-b) The expression levels of XIST and miR-30d-5p in NG or HG-treated MPC5 cells were determined by qRT-PCR. GAPDH or U6 was used as the internal control. (c–e) The expressions of XIST, miR-30d-5p, and BECN-1 in MPC5 cells transfected with XIST overexpression plasmid or empty vector were determined by qRT-PCR. GAPDH or U6 was used as the internal control. (f-g) The expressions of miR-30d-5p and BECN1 in MPC5 cells transfected with miR-30d-5p mimic/mimic control were determined by qRT-PCR. GAPDH or U6 was used as the internal control. (h–j) The first two groups of MPC5 cells were treated with NG or HG. The last four groups of MPC5 cells cotransfected with XIST overexpression plasmid/empty vector and miR-30d-5p mimic/mimic control were treated with HG. (h) The viability of MPC5 cells was determined by CCK-8 assay. (i-j) The apoptosis of MPC5 cells was determined by flow cytometry. Quantified values were presented as mean ± standard deviation of at least three independent experiments. ^+++^*p* < 0.001 vs. vector. ^△△△^*p* < 0.001 vs. MC. ^*∗∗*^*p* < 0.001 vs. NG.  ^*p* < 0.05, ^  ^  ^*p* < 0.001 vs. vector + MC. ^###^*p* < 0.001 vs. XIST + MC. ^&&^*p* < 0.01, ^&&&^*p* < 0.001 vs. vector + M. HG: high glucose. NG: normal glucose. M: mimic. MC: mimic control. qRT-PCR: quantitative real-time polymerase chain reaction. GAPDH: glyceraldehyde-3-phosphate dehydrogenase; XIST: X inactive specific transcript.

In addition, the expression of XIST was lower, and the expression of miR-30d-5p was higher in HG-induced MPC5 cells (Figures 3(a) and 3(b), *P* < 0.001). We constructed XIST overexpression plasmid and verified its transfection efficiency (Figure 3(c), *P* < 0.001), and found that OE-XIST can reduce the level of miR-30d-5p while increasing the level of BECN1 (Figures 3(d) and 3(e), *P* < 0.001). At the same time, we also tested the transfection efficiency of miR-30d-5p mimic in MPC5 cells and found that miR-30d-5p mimic increased the miR-30d-5p expression while reducing the expression of BECN1 (Figures 3(f) and 3(g), *P* < 0.001). Subsequently, we observed changes in the biological behaviors of the MPC5 cells. Under HG induction, the viability of MPC5 cells was decreased, while the apoptosis was increased (Figures 3(h)–3(j), *P* < 0.001). Additionally, OE-XIST promoted the viability and suppressed the apoptosis of HG-induced MPC5 cells, whereas miR-30d-5p mimic did the opposite (Figures 3(h)–3(j), *P* < 0.001). The aforementioned effects of OE-XIST or miR-30d-5p mimic were reversed by cotransfection of OE-XIST and miR-30d-5p mimic (Figures 3(h)–3(j), *P* < 0.01).

### 3.4. XIST Targeted miR-30d-5p to Regulate the Expressions of Apoptosis-, EMT- and Autophagy-Related Proteins

**Figure 4:**
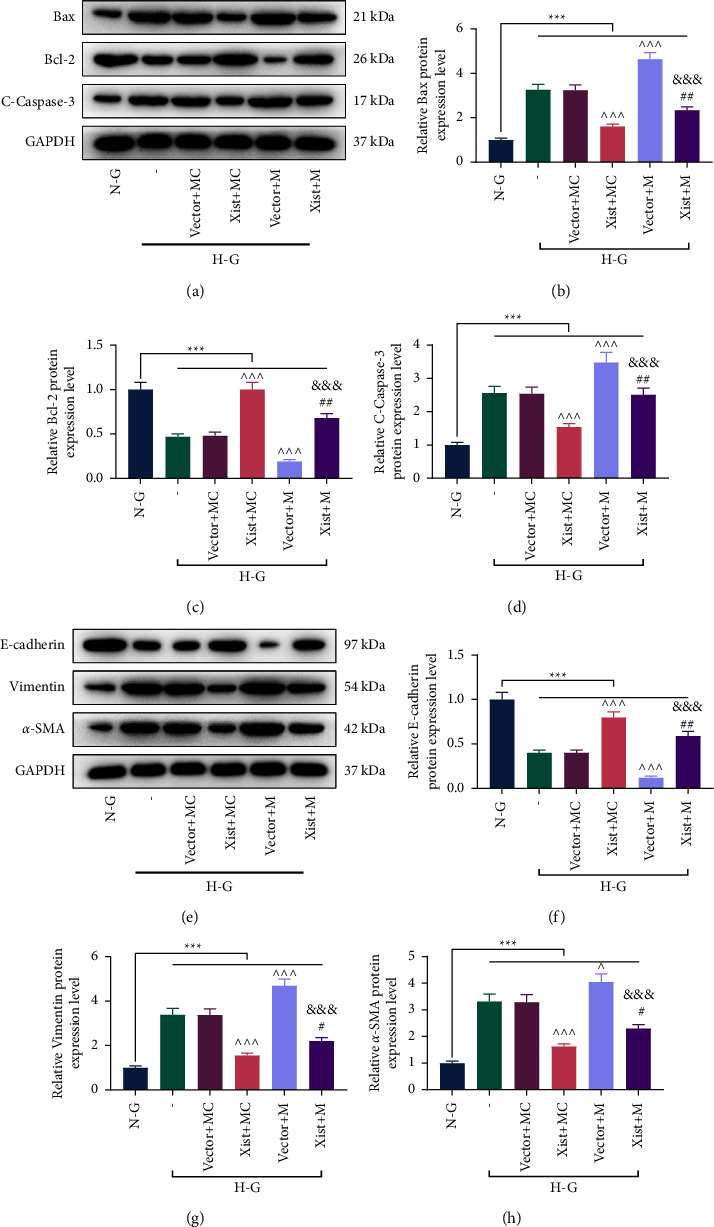
XIST targeted miR-30d-5p to regulate apoptosis- and EMT-related proteins in HG-induced podocytes. The experiments were divided into six groups. The first two groups of MPC5 cells were treated with NG or HG. The last four groups of MPC5 cells cotransfected with XIST overexpression plasmid/empty vector and miR-30d-5p mimic/mimic control were treated with HG. (a–d) The expressions of apoptosis-related proteins (Bax, Bcl-2, and cleaved caspase-3) in MPC5 cells were determined by Western blot. GAPDH was used as the internal control. (e–h) The expressions of EMT-related proteins (E-cadherin, Vimentin, and *α*-SMA) in MPC5 cells were determined by Western blot. GAPDH was used as the internal control. Quantified values were presented as mean ± standard deviation of at least three independent experiments.  ^∧^*p* < 0.05,  ^∧ ∧ ∧^*p* < 0.001 vs. vector + MC. ^#^*p* < 0.05, ^##^*p* < 0.01, ^###^*p* < 0.001 vs. XIST + MC. ^&&^*p* < 0.01, ^&&&^*p* < 0.001 vs. vector + M. HG: high glucose. NG: normal glucose. M: mimic. MC: mimic control. EMT: epithelial-mesenchymal transition. GAPDH: glyceraldehyde-3-phosphate dehydrogenase; XIST: X inactive specific transcript.

**Figure 5:**
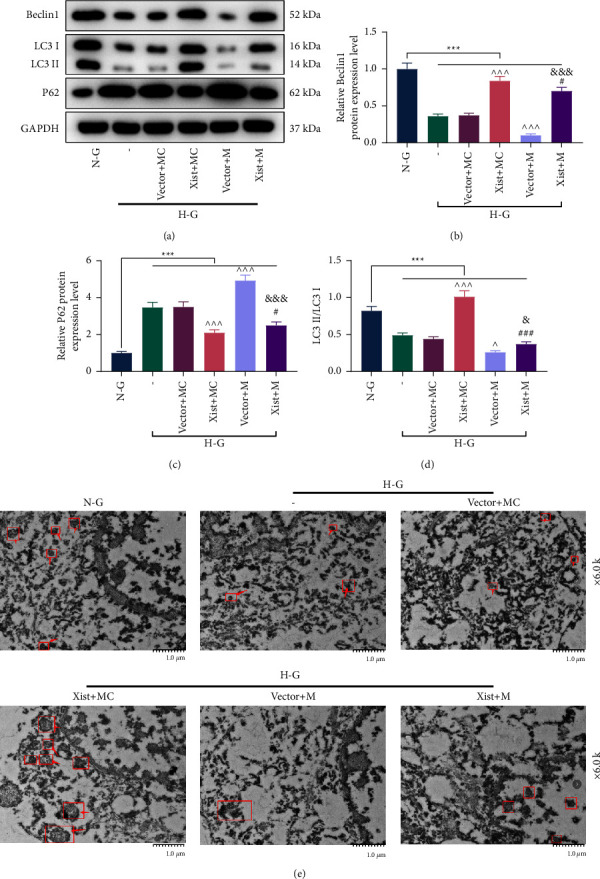
XIST targeted miR-30d-5p to regulate autophagy-related proteins and autophagosomes in HG-induced podocytes. The experiments were divided into six groups. The first two groups of MPC5 cells were treated with NG or HG. The last four groups of MPC5 cells cotransfected with XIST overexpression plasmid/empty vector and miR-30d-5p mimic/mimic control were treated with HG. (a–d) The expressions of autophagy-related proteins (Beclin-1, LC3I/II, and *P*62) in MPC5 cells were determined by Western blot. GAPDH was used as the internal control. (e) The autophagosomes in MPC5 cells were observed by the transmission electron microscope (magnification: 6,000x times; scale bar = 1 *μ*m). The quantified values were presented as mean ± standard deviation of at least three independent experiments. ^∧^*p* < 0.05, ^∧ ∧ ∧^*p* < 0.001 vs. vector + MC. ^#^*p* < 0.05, ^###^*p* < 0.001 vs. XIST + MC. ^&^*p* < 0.001, ^&&&^*p* < 0.001 vs. vector + M. HG: high glucose. NG: normal glucose. M: mimic. MC: mimic control. GAPDH: glyceraldehyde-3-phosphate dehydrogenase; XIST: X inactive specific transcript.

We evaluated the regulation of XIST and miR-30d-5p on apoptosis-, EMT-, and autophagy-related proteins. HG-induced upregulation of Bcl-2-associated X protein (Bax), cleaved caspase-3, vimentin, alpha-smooth muscle actin (*α*-SMA), and *P*62 in MPC5 cells, as well as downregulation of B-celllymphoma-2 (Bcl-2), E-cadherin, Beclin-1, and LC3 II/I in MPC5 cells (Figures 4(a)–4(h) and 5(a)–5(d), *P* < 0.001). However, these effects induced by HG were negated by OE-XIST and enhanced by miR-30d-5p mimic (Figures 4(a)–4(h) and 5(a)–5(d), *P* < 0.05). Nevertheless, the aforementioned effects of OE-XIST or miR-30d-5p mimic were countervailed by cotransfection of OE-XIST and miR-30d-5p mimic (Figures 4(a)–4(h) and 5(a)–5(d), *P* < 0.05). In addition, HG treatment reduced the number of autophagosomes in MPC5 cells, which were neutralized by OE-XIST (Figure 5(e)). Moreover, miR-30d-5p mimic reduced the number of autophagosomes in HG-induced MPC5 cells and offset the effects of OE-XIST (Figure 5(e)).

## 4. Discussion

Inducing autophagy to protect podocytes from HG-induced injury has become a promising strategy in the treatment of DN [17]. Under normal blood glucose levels, autophagy is an important protective mechanism in renal epithelial cells, including podocytes, proximal tubular, mesangial, and endothelial cells; under hyperglycemic conditions, repression of the autophagic mechanism can contribute to the development and progression of diabetic kidney disease [18, 19]. In this study, we clarified that the mechanism of the XIST/miR-30d-5p/BECN1 axis in protecting podocytes from HG-induced injury is related to the promotion of podocyte autophagy.

The role of XIST in DN is various. Wang reported that XIST facilitates the development of DN by affecting the biological behaviors of human mesangial cells and regulating inflammation [20]. Yang et al. pointed out that XIST facilitates renal interstitial fibrosis in DN by upregulating the expressions of fibrosis-associated markers in HK-2 cells exposed to high glucose [21]. Meanwhile, Long et al. found that XIST expression is downregulated in HG-induced podocytes, and XIST overexpression promotes viability while inhibiting the apoptosis of podocytes [13]. Consistent with those of Long et al., our results uncovered that XIST overexpression has a protective effect on podocytes induced by HG. However, this result seems to be contrary to the conclusion reported by Wang [20] and Yang et al. [21] that XIST promotes the development of DN. This contradictory phenomenon may be due to the different cells studied or the different functions of XIST as lncRNA [22].

The relationship between XIST and autophagy has been reported in a variety of cells, such as hepatic stellate cells [23] and ovarian cancer cells [24]. Overexpression of XIST increases the level of LC3II/LC3I yet decreases the level of *p*62 to induce autophagy in nucleus pulposus cells [25]. XIST knockdown weakens the proliferation and autophagy in retinoblastoma cells [26]. In this study, we also found that XIST overexpression increased the LC3II/LC3I and Beclin1 levels but decreased *p*62 protein level to activate the autophagy of HG-induced podocytes. In addition, the targeting relationship between XIST and miR-30d-5p has been verified in rat Schwann cells [27], and it has also been proved that XIST overexpression can reduce diabetic peripheral neuropathy by repressing miR-30d-5p expression to induce autophagy [27]. In addition, the inhibitory effect of miR-30d-5p on autophagy has been demonstrated in renal cell carcinoma and hypoxic-ischemic rats [12, 28]. Analogous to previous studies, we found that upregulation of miR-30d-5p reversed the regulation of XIST overexpression on HG-induced podocyte autophagy, indicating that the protective effect of XIST on podocyte injury was achieved by targeting miR-30d-5p.

Classical autophagy is divided into five stages, including the initiation process, phagocyte nucleation, phagocyte expansion, autophagosome-lysosome fusion, and lysosomal substrate degradation [29]. Beclin1 is involved in the formation of autophagic precursors, and LC3-I can bind to *P*62 after being cleaved into LC3-II, mediating the aggregation of ubiquitinated proteins, followed by *P*62 degradation with the ubiquitinated proteins [29]. Therefore, increasing the protein expression of Beclin 1 is beneficial to the promotion of autophagy. In this study, we found that BECN1 was the target gene of miR-30d-5p. Fengyan Zhao et al. found that miR-30d-5p antagonist could upregulate the Beclin1 level, promote autophagy, and then restore the neurological function of hypoxic-ischemic rats [28]. A study on DN showed that HG-induced downregulation of Beclin-1 and LC3 II/I as well as upregulation of *P*62 in MPC5 [30]. In our research, the same phenomenon was observed, but the effect of HG was nullified by XIST overexpression and potentiated by miR-30d-5p upregulation, indicating that XIST boosted the expression of BECN1 by inhibiting miR-30d-5p, thus promoting autophagy.

Podocyte apoptosis is one of the main causes of the decrease in the number of podocytes, and there is a crosstalk between autophagy and apoptosis of podocytes [31]. It is reported that autophagy can protect the podocytes from apoptosis [32]. Silencing SPAG5-AS1 promotes autophagy yet inhibits the apoptosis of podocytes via SPAG5/AKT/mTOR pathway to alleviate podocyte injury [33]. Jin et al. revealed that exosomes derived from adipose-derived stem cells alleviate diabetic nephropathy by promoting autophagy flux and attenuating apoptosis in podocytes [34]. Liu et al. proved that XIST targets miR-30d-5p to facilitate autophagy and thus inhibit the apoptosis of Schwann cells [27]. In our study, XIST overexpression impeded podocyte apoptosis, while miR-30d-5p reversed the effect of XIST overexpression, suggesting that XIST promoted the autophagy of podocytes by downregulating miR-30d-5p to hamper the podocyte apoptosis.

HG can induce podocyte EMT in the process of DN [35]. In this process, the great loss of E-cadherin (the landmark molecule of epithelial cells), together with abundant expression levels of vimentin and *α*-SMA (the landmark molecules of interstitial cells), can be observed in podocytes, which leads to the increased fusion of podocytes and the decreased adhesion to the glomerular basement membrane, finally resulting in decreased podocytes [36]. However, EMT is a reversible process, so DN can be alleviated by inhibiting the EMT of podocytes [37]. In addition, Shi et al. found that the EMT of podocytes exposed to HG can be reduced by restoring autophagy activity [38]. Consistent with previous research results, we discovered that XIST promoted the autophagy of podocytes by modulating the miR-30d-5p/BECN-1 axis, and then inhibited EMT of podocytes.

In summary, our data indicate that XIST promotes the autophagy of podocytes by regulating the miR-30d-5p/BECN1 axis, thereby protecting podocytes from HG-induced injury. This study provides a new target for the treatment of DN.

## Figures and Tables

**Table 1:** Antibodies used in this study.

Name	Catalog	Molecular weight	Dilution	Manufacturer
Beclin 1	ab210498	52 kDa	1/1000	Abcam, UK
LC3 I/II	ab192890	16/14 kDa	1/2000	Abcam, UK
*P*62	ab109012	62 kDa	1/10000	Abcam, UK
Bax	ab32503	21 kDa	1/1000	Abcam, UK
Bcl-2	ab182858	26 kDa	1/2000	Abcam, UK
Cleaved caspase-3	ab231289	17 kDa	1/1000	Abcam, UK
E-cadherin	ab231303	97 kDa	1/1000	Abcam, UK
Vimentin	ab92547	54 kDa	1/1000	Abcam, UK
*α*-SMA	ab124964	42 kDa	1/1000	Abcam, UK
GAPDH	ab8245	37 kDa	1/10000	Abcam, UK
Goat antirabbit	ab205718	—	1/2000	Abcam, UK
Goat antimouse	ab205719	—	1/2000	Abcam, UK

LC3 I/II, light chain 3 I/II; Bax, Bcl-2-associated X protein; Bcl-2, B-celllymphoma-2;*α*-SMA, alpha-smooth muscle actin; GAPDH, Glyceraldehyde-3-phosphate dehydrogenase.

**Table 2:** Primers used in this study.

Genes	5′ --> 3′
XIST forward	ACGCTGCATGTGTCCTTAG
XIST reverse	GAGCCTCTTATAAGCTGTTTG
miR-30d-5p forward	TGTAAACATCCCCGAC
miR-30d-5p reverse	GTGCAGGGTCCGAGGT
Becn1 forward	GCCTCTGAAACTGGACACGA
Becn1 reverse	TAGCCTCTTCCTCCTGGGTC
GAPDH forward	CCCTTAAGAGGGATGCTGCC
GAPDH reverse	ACTGTGCCGTTGAATTTGCC
U6 forward	CTCGCTTCGGCAGCACA
U6 reverse	ACGCTTCACGAATTTGCGT

XIST, X inactive specific transcript; GAPDH, glyceraldehyde-3-phosphate dehydrogenase.

## Data Availability

The analyzed data sets generated during the study are available from the corresponding author upon reasonable request.
